# A non-toxic approach for treatment of breast cancer and its metastases: capecitabine enhanced photodynamic therapy in a murine breast tumor model

**DOI:** 10.20517/2394-4722.2018.98

**Published:** 2019-01-24

**Authors:** Sanjay Anand, Anton Yasinchak, Taylor Bullock, Mukul Govande, Edward V. Maytin

**Affiliations:** 1 Department of Biomedical Engineering, Lerner Research Institute, Cleveland Clinic, Cleveland, OH 44195, USA.; 2 Institute of Plastic Surgery and Dermatology, Cleveland Clinic, Cleveland, OH 44195, USA.; 3 Department of Molecular Medicine, Cleveland Clinic Lerner College of Medicine, Cleveland Clinic, Cleveland, OH 44195, USA.

**Keywords:** Photodynamic therapy, protoporphyrin IX, aminolevulinic acid, breast cancer, metastasis, cell death, 4T1, *in vivo* imaging system

## Abstract

**Aim:**

Breast cancer (BCA) in women is a leading cause of mortality and morbidity; distant metastases occur in ~40% of cases. Here, as an alternative to ionizing radiation therapy and chemotherapy and their associated side effects, we explored a new combination approach using capecitabine (CPBN) and aminolevulinate-based photodynamic therapy (PDT). We had previously developed a combination PDT approach in which 5-fluorouracil (5FU), a differentiation-promoting agent, increases the levels of protoporphyrin IX (PpIX) in cancer cells when given as a neoadjuvant prior to aminolevulinic acid (ALA). However, 5FU can be toxic when administered systemically at high levels. We reasoned that CPBN, a known chemotherapeutic for BCA and less toxic than 5FU (because CPBN is metabolized to 5FU specifically within tumor tissues), might work equally well as a PDT neoadjuvant.

**Methods:**

Murine 4T1 BCA cells harboring a luciferase transgene were injected into breast fat pads of female nude mice. CPBN (600 mg/kg/day) was administered by oral gavage for 3 days followed by intraperitoneal ALA administration and PDT with red light (633 nm) on day 4. Tumor growth and regression were monitored *in vivo* using bioluminescence imaging. Histological changes in primary tumors and metastases were assessed by immunohistochemistry after necropsy.

**Results:**

CPBN pretreatment of 4T1 tumors increased cellular differentiation, reduced proliferation, raised PpIX levels, enhanced tumor cell death, and reduced metastatic spread of 4T1 cells post-PDT, relative to vehicle-only controls.

**Conclusion:**

The use of CPBN as a non-toxic PDT neoadjuvant for treatment of BCA represents a novel approach with significant potential for translation into the clinic.

## INTRODUCTION

Breast cancer (BCA) is the most common type of malignancy in women, with 1.7 million new cases diagnosed worldwide each year^[1]^ and it is also a leading cause of mortality among women, accounting for over half a million deaths annually^[1–3]^. The main cause of death is not the primary tumor, but distant metastases that create significant morbidity. Although 80% of BCA patients receive adjuvant chemotherapy, ~40% of those patients relapse and ultimately die due to metastatic disease^[4]^. While 5-year survival rates of BCA patients are increasing overall due to advanced therapeutic techniques, relatively low survival rates and poor prognoses are still observed in patients with metastatic BCA^[4]^. BCA cells metastasize mainly to the lung, liver, brain and bone; metastases to the skin (cutaneous metastasis) occur in ~20% of cases^[4]^. Local relapse and chest wall metastases occur at a rate of 5%^[4]^. The chest wall tumors are notable because they can be very painful, and are hard to treat due to their resistance to chemotherapy^[5,6]^. Ionizing radiation therapy (RT) has been successfully used for the treatment of cutaneous chest wall metastases; however, multiple rounds of therapy are required and are typically associated with side effects such as radiation dermatitis, blistering and chronic ulcers. An alternative to RT, constituting a safer treatment that might be given either alone or together with chemotherapy, is urgently needed.

Photodynamic therapy (PDT) is a non-mutagenic, non-scarring treatment modality for cancer. PDT employs a photosensitizer (PS) and visible light in the presence of oxygen to kill tumor cells^[7–9]^. During PDT, two steps provide dual selectivity. First, the cancer cells accumulate and retain PS to a greater extent than normal cells. Second, focusing the light source onto the tumor further enhances tumor specificity. The PS is activated, triggering cell death by releasing free radicals^[8]^. In the early history of PDT, the PS was given systemically and carried a high risk of increased skin phototoxicity (sunburn). To reduce toxicity and improve tumor selectivity, a newer mechanism of PDT that uses a prodrug, aminolevulinic acid (ALA) instead of the pre-formed PS, was developed^[10,11]^. ALA, given systemically, orally or topically, is taken up and enzymatically converted into protoporphyrin IX (PpIX) within mitochondria. PpIX is then activated by strong visible light to generate reactive oxygen species that kill the cancer cells^[9,12,13]^. Skin cancers are easily illuminated from the surface using red light that penetrates up to 1 cm into tissue. PDT is now successfully used in Europe to treat superficial basal cell carcinoma and squamous cell carcinoma (SCC)^[14]^. PDT has several advantages over other modalities: (1) unlike surgical excision, PDT is non-scarring (and may actually inhibit fibrosis^[15,16]^); and (2) unlike RT, PDT can be repeated multiple times and because PDT targets mitochondria rather than DNA, there is minimal risk of genetic mutations.

Although PDT is currently employed mostly for skin cancers, it carries tremendous potential to shrink or eliminate BCA and its associated metastases; this research area has only been explored in a preliminary fashion^[17,18]^. Up until now, the need for specialized equipment and better-standardized protocols has delayed the acceptance of PDT as a main line of treatment for BCA and its metastases. In a recent clinical trial in China, ALA-PDT used in combination with RT to treat cutaneous BCA metastases, showed a better complete response (50% *vs.* 20%) and a reduced time to clearance (110 days *vs.* 175 days) *vs.* RT alone^[19]^. It was also shown that lowering the photosensitizing drug dose and the light fluence rate can improve the clearance of BCA chest wall lesions^[19]^. Here, we seek to apply a different combination therapy PDT approach to BCA. Using pre-clinical murine models and subsequent clinical trials in patients with skin cancer, we have established that certain neoadjuvantal agents can significantly improve PpIX levels and distribution within tumors when combined with ALA-PDT^[9,20]^. In this study, we explore an approach that combines two FDA-approved drugs [capecitabine (CPBN) and ALA] and a standard light source used in dermatology, to deliver a safer, more efficacious, and more convenient treatment for localized BCA tumors and metastases in mice.

The rationale for our approach is based upon several relatively new scientific principles. In the past, several PDT regimens have been explored with different PS molecules, routes and doses of PS, and various wavelengths, illumination protocols and oxygen concentrations (hypoxia *vs.* normoxia), in attempts to improve PDT responses^[7–9]^. However, since cell physiology (particularly the state of tumor differentiation) plays an important role in PDT outcome, we considered biomodulation with neoadjuvants given prior to PDT as another avenue for optimization^[9]^. Towards this goal, we pioneered a concept called “differentiation-enhanced” or “combination PDT”. Thus, cancer cells of different tissue origins, when pre-treated with differentiation promoting agents, show elevated accumulation of PpIX and enhanced cell death after ALA-PDT^[9]^. Three such agents, methotrexate^[21,22]^, vitamin D (calcitriol)^[23]^ and 5-fluorouracil (5-FU)^[24]^ were shown to improve tumor responses in skin, prostate and BCA models when used as neoadjuvants for PDT. Pretreatment with any of these agents resulted in 3- to 5-fold upregulation of mitochondrial PpIX production^[9]^. The effects on PpIX levels were selective for tumor cells and did not occur in adjacent skin^[24,25]^. We recently worked out the molecular mechanisms for this effect. Coproporphyrinogen oxidase, a heme enzyme located upstream of PpIX, is upregulated; ferrochelatatse located downstream of PpIX, is decreased; and the net effect favors PpIX buildup^[23,26]^. An important feature of these neoadjuvants is that they drive cancer cells toward a more highly differentiated state, as determined by the elevated expression of E-cadherin in tumors. Using a BCA model (MDA-MB-231 cells implanted in breast fat pads of nude mice), we showed that pretreatment with calcitriol (the active form of Vit D) prior to ALA-PDT resulted in an enhanced therapeutic response relative to ALA-PDT alone^[25]^. Using topical 5-FU, we recently completed a clinical trial in patients with actinic keratosis (AK; pre-SCC), and showed that combination PDT with topical 5-FU significantly enhanced the PDT efficacy as determined by increased clearance of the AK lesions^[20]^.

A major reason for PDT treatment failure is the heterogeneity of PpIX distribution within the target tissue. Some microanatomical pockets, located deep inside the tumor, produce minimal PpIX due to poor PS penetration and suboptimal physiological response, thereby escaping PDT photodamage^[26]^. When 5-FU is given as a neoadjuvant prior to ALA, PpIX levels are raised in the vast majority of tumor cells, resulting in enhanced cell death following PDT in both skin tumors and in murine breast tumors^[24]^. However, the systemic dose of 5-FU required to achieve this effect is quite high and might be toxic in humans. In the current study, in order to circumvent toxicity, we used an alternative drug that is FDA-approved for treatment of metastatic BCA^[27–29]^. CPBN is a precursor form of 5-FU that is selectively converted into the active end product, 5-FU, primarily in cancer cells. CPBN is converted to 5-FU in three enzymatic steps requiring carboxylesterase, cytidine deaminase (CDA), and thymidine phosphorylase (TYMP)^[28,30]^. Cancer cells have relatively high levels of CDA and TYMP, explaining the increased sensitivity of cancer cells to CPBN, and the low adverse effects of this drug on non-tumoral tissues^[28]^. To test and develop a combination approach with CPBN and PDT, we used a mouse BCA model (the 4T1 cell line; a triple-negative BCA equivalent to human stage IV) that has already been previously utilized in many studies of BCA^[31,32]^. The long-term goal of our study was to develop a better mechanistic understanding of the CPBN-PDT approach, for possible translation of this concept to the clinic for treating localized BCAs.

## METHODS

### Cell culture

4T1, a murine breast carcinoma line (a triple-negative breast cancer and stage IV human BCA equivalent) and Bioware^®^ Brite Cell Line 4T1-Red-FLuc, were purchased from animal type culture collection and Perkin Elmer Inc., respectively, and cultured as per the instructions provided by the source/vendor.

### 4T1 murine breast tumor model

Murine 4T1 cells (0.5 × 10^6^) and 4T1-Red-Fluc cells (0.005 × 10^6^) were resuspended in the growth medium and injected into breast fat pads of female nude mice. Visible/palpable tumors developed in 3–5 days post injection. All experimental procedures involving mice were preapproved by the Institutional Animal Care and Use Committee of the Cleveland Clinic.

### Pretreatment with CPBN

CPBN (Sigma Aldrich, 600 mg/kg/day) was dissolved in 5% gum arabic-sodium citrate solution (vehicle) and was given by oral route (gavage) once daily for 3 days. Control mice received vehicle solution only. On day 4, ALA was administered through intraperitoneal route (IP; 200 mg/kg in phosphate buffered saline) for 4 h and the mice were either exposed to red light (633 nm) for PDT, or euthanized for tumor harvest and histological and immunohistochemical analyses^[23,24]^. The dose of oral CPBN chosen for our study (600 mg/kg/day for three days) was based upon 80% of the maximum tolerated dose determined in nude mice by Kolinsky *et al.*^[33]^, maximum tolerated dose 700 mg/kg/day when given for 7 days. Note that dose translation between CPBN (Xeloda^®^) doses used in humans^[34,35]^ and biologically equivalent doses in mice is not straightforward, and typically involves body surface normalization methods^[36,37]^.

### Imaging of tumor growth/regression and metastases using *in vivo* imaging system (IVIS^®^ spectrum)

4T1 tumor bearing mice were injected with *D*-luciferin (IP; 150 mg/kg; Perkin Elmer Inc.) and imaged under continuous isoflurane anesthesia using the *in vivo* imaging system (IVIS spectrum; Perkin Elmer Inc.) following the manufacturer’s instructions. Bioluminescence imaging signals were recorded as bioluminescence units (BLUs, photons/s/cm^2^/sr), and used to analyze tumor growth/regression and metastases to distant sites using Live Imaging Software^[25]^. Digital images were captured 5 min after *D*-luciferin injection, along with a radiance calibration standard scale, at each weekly time point during tumor growth/regression experiments. Tumor-associated signals (emitted light units) from each region of interest (ROI) were normalized per this radiance standard scale.

### Treatment with PDT

Mice were anesthetized using intraperitoneal ketamine and xylazine. Anesthetized mice with 4T1 tumors (± CPBN pretreatment and after 4 h of ALA), were exposed to 100 J/cm^2^ of 633 nm light using a LumaCare^®^ xenon source (LumaCare). The light source was calibrated using a FieldMate^®^ laser power meter (Coherent). Mice were either imaged at different time points to analyze the therapeutic response and metastases to distant sites or sacrificed and tumors harvested at 24 h post PDT for histological and immunohistochemical analyses^[23,24]^.

### Imaging of PpIX in 4T1 tumors by confocal microscopy

Tumor cryosections (10 mm) were placed on glass slides, briefly air dried, mounted under coverslips with Vectashield (Vector Laboratories), and viewed on a confocal microscope (Leica Microsystems; 40′ magnification; excitation at 635 nm and image collection at 650–780 nm). Quantitation of relative PpIX levels from digital photomicrographs was performed using IP Lab software^[23,24]^.

### Histology, immunohistochemistry and cell death analyses

The 4T1 tumors and distant metastatic sites were harvested at different time points, fixed in formalin, paraffin-embedded and sectioned (5 mm) following a standard protocol. Hematoxylin and eosin staining, immunofluorescence staining, and cell death analysis [terminal-deoxynucleoitidyl transferase dUTP nick end labeling (TUNEL)] were performed as described^[23,24]^.

### Statistical analyses

Statistical analyses were performed using a two-sided *t*-test to compare differences between PpIX levels, expression of markers by immunohistochemistry, cell death and tumor bioluminescence. *P* ≤ 0.05 was considered statistically significant.

## RESULTS

Experiments were designed to test our hypothesis that oral CPBN, given as a differentiation-promoting agent (instead of administering its final product 5-FU{Anand, 2017 #100;Maytin, 2018 #114}), may enhance the PS levels, subsequent cell death response and will impact distant metastases following ALA-PDT in a murine 4T1 breast tumor model.

### Combination of oral CPBN before ALA enhances PpIX levels in 4T1 tumors

**Figure 1.**
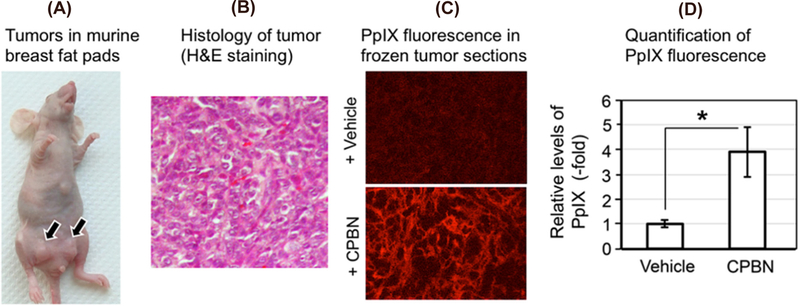
Analyses of protoporphyrin IX (PpIX) in murine breast tumor model. (A) Photograph of a nude female mouse with 4T1 subcutaneous tumors in breast fat pad; (B) hematoxylin and eosin (H&E) stained 4T1 tumor section showing histological details of the murine breast carcinoma shown in (A); (C) confocal micrographs of frozen sections of 4T1 tumors after oral treatment with vehicle (top) or capecitabine (CPBN) (bottom) followed by aminolevulinic acid; red signal is mitochondrial PpIX; (D) quantitation of PpIX-specific fluorescence from confocal micrographs shown in (C); integrated fluorescence intensity was measured using IPLab software. Mean SEM (18 images from 6 tumors) from three independent experiments is shown. *P = 0.0001

To study if oral CPBN can successfully replace its relatively toxic final product 5-FU for the purpose of promoting differentiation prior to ALA-PDT, a murine breast tumor cell line (4T1) equivalent to stage IV human triple negative breast carcinoma was injected in the breast fad pad of female nude mice to generate a murine breast tumor model [Figure 1A and B]. Following the injection of 4T1 cells, mice with visible and palpable 4T1 breast tumors [Figure 1A] were given either CPBN or vehicle (orally by gavage, once a day for 3 days), followed by intraperitoneal ALA administration on the 4th day. Tumors were harvested and frozen sections were analyzed by confocal microscopy using excitation and emission settings that allow visualization of PpIX-specific fluorescence. In the 4T1 tumors from mice treated with oral CPBN, PpIX was more abundant and also present throughout the tumor at much higher levels, as compared with vehicle treated (control) tumors [Figure 1C; bottom *vs.* Top]. Digital quantification of the PpIX fluorescence signal from confocal images confirmed this observation by showing a statistically significant increase of ~4-fold in PpIX levels in CPBN treated tumors [Figure 1D].

### CPBN pretreatment enhances differentiation, inhibits proliferation and enhances cell death following PDT

**Figure 2.**
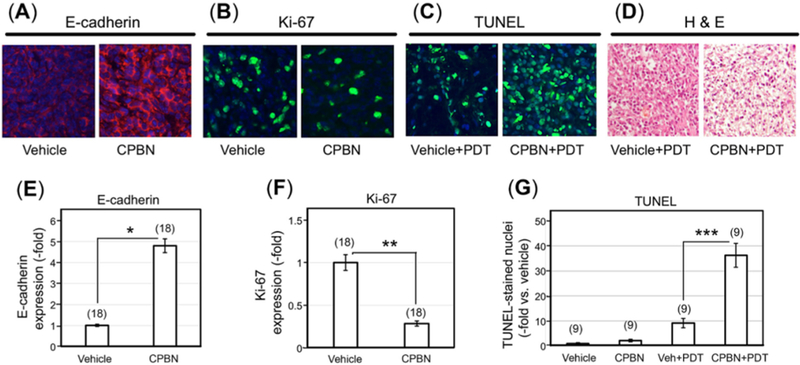
Physiological and cell death responses to capecitabine pretreatment and photodynamic therapy in murine breast tumor model. Photomicrographs showing 4T1 tumor sections immunohistochemically stained for (A) E-cadherin (E-cad), a marker of differentiation; (B) Ki67, a marker of proliferation, following vehicle or capecitabine (CPBN) treatment; (C) Cell death analysis by terminal-deoxynucleoitidyl transferase dUTP nick end labeling (TUNEL) labeling of apoptotic nuclei at 24 h post photodynamic therapy (PDT), following vehicle or CPBN pre-treatment. Nuclei in images shown in (A-C) were stained with 4′,6-diamidino-2-phenylindole (DAPI); (D) hematoxylin and eosin (H&E) staining of tumor sections showing enhanced cell death at 24 h post PDT in tumors pretreated with CPBN or vehicle alone. Graphs show quantitation of changes in (E) E-cadherin signal; (F) Ki67 signal; and (G) TUNEL positive nuclei per high power field. All graphs show Mean ± SEM from multiple images (number of images shown in parentheses) from three independent experiments. *P* values from an unpaired two-sided *t* -test are shown: *P = 1.57 × 10^−11^; **P = 1.20 × 10^−8^; ***P = 6.02 × 10^−9^

5FU, a chemotherapeutic drug and the final product of CPBN metabolism specifically converted in the tumors due to high levels of CDA, and TYMP^[28,30]^, is known to downregulate DNA synthesis through inhibition of thymidylate synthase (TS) and *de novo* synthesis of thymidylic acid^[38]^. In addition to its anti-proliferative effect, 5-FU induces epithelial cell differentiation^[9]^. The physiological effects of CPBN pretreatment on the 4T1 tumors were analyzed by immunostaining of tumor sections for expression of E-cadherin and Ki67, markers of differentiation and proliferation, respectively [Figure 2]. As anticipated, relative to vehicle treated tumors, CPBN pretreatment led to a 5-fold increase in differentiation [E-cadherin expression; Figure 2A and E]. In the same set of tumors, CPBN pretreatment resulted in a 70% reduction in cell proliferation [Ki67 expression; Figure 2B and F].

To assess the effects of combination therapy upon 4T1 intratumoral cell death, CPBN + PDT treated tumors along with vehicle- and CPBN-treatment controls were harvested 24 h after PDT and histological sections were analyzed by TUNEL assay and by hematoxylin and eosin staining. TUNEL is a technique in which cleaved inter-nucleosomal DNA fragments are enzymatically labeled in dying cells. CPBN pretreated tumors showed an approximately 4-fold increase in TUNEL positive nuclei, relative to their vehicle treated PDT controls by 24 h post PDT [Figure 2C and G]. Histomorphological details of CPBN-PDT induced cell death in 4T1 tumors were analyzed by comparing hematoxylin and eosin stained sections from tumors treated with CPBN + PDT or with PDT alone [Figure 2D]. At 24 h following PDT, all tumors displayed numerous apoptotic cells with shrunken, pyknotic nuclei, intracellular vacuoles, and large areas of cell loss (empty spaces), but these features were more pronounced in the CPBN pretreated tumors, similar to quantitation of cell death features by hematoxylin and eosin reported previously^[25,39]^.

### Non-invasive visualization and monitoring of tumor growth and regression after PDT using IVIS

**Figure 3.**
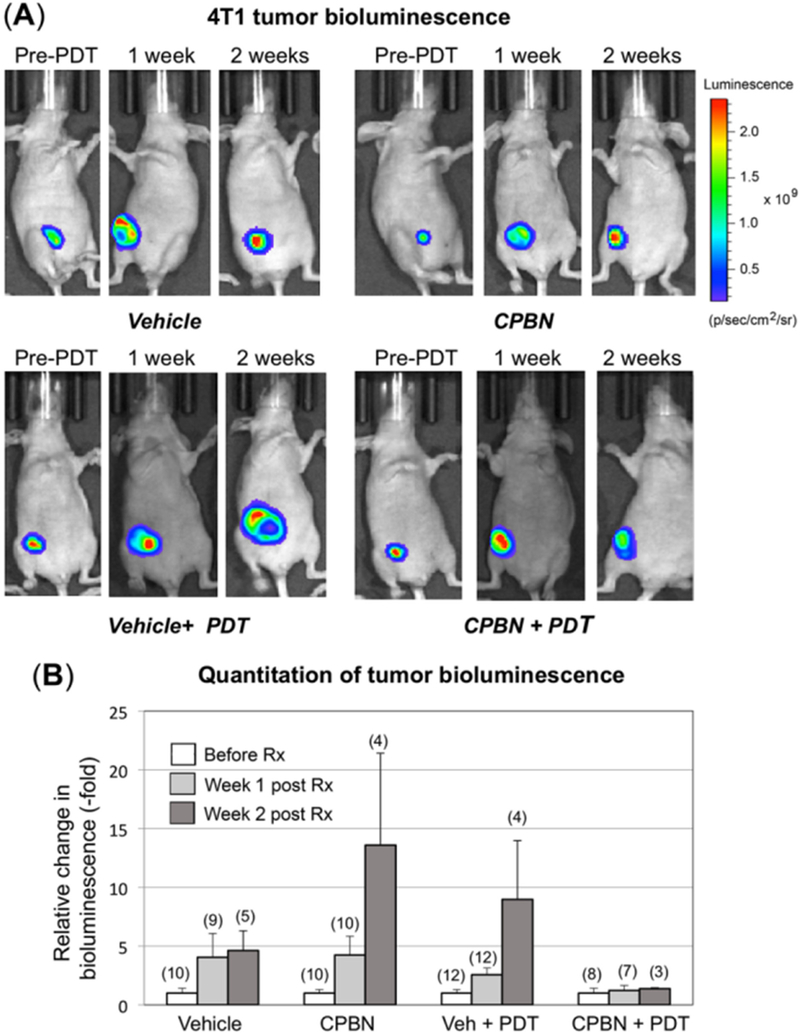
Monitoring of therapeutic responses to capecitabine and/or photodynamic therapy (PDT) in murine breast tumor model using an in vivo imaging system (IVIS). 4T1 murine breast cancer cells (stably transfected with luciferase) were implanted in the breast fat pad of nude mice and monitored for bioluminescence signals originating from tumors, using an IVIS instrument. (A) Tumor growth or shrinkage after 1 or 2 weeks of vehicle- or capecitabine (CPBN)-only, ALA + PDT or CPBN + PDT, was monitored by injection of luciferin, and measurement and quantitation of luminescence output using Live imaging software as described^[25]^. A typical radiance scale, common to most of the bioluminescent images, is shown in (A); (B) quantitation of bioluminescent light units, relative to pre-PDT tumor luminescence from each treatment group, is shown as fold increase. Bars representing mean (−/+ SEM), from the number of mice shown in parentheses on top of the bars (pooled from four independent experiments) are shown. The differences observed in the relative bioluminescence between vehicle + PDT and CPBN + PDT at one (*P* = 0.14) and two (*P* = 0.20) weeks post PDT, were not statistically significant

Using the IVIS Spectrum^®^ device, growth and/or regression of orthotopic 4T1 breast tumors in nude mice from different treatment groups (vehicle, CPBN, vehicle + PDT and CPBN + PDT) was monitored non-invasively. A genetically modified version of 4T1 cells (4T1-red-fluc Bioware^®^ Brite cell line), which had been stably transduced with the red-shifted firefly luciferase gene from Luciola Italica (Red-FLuc), was used to monitor the effect of vehicle or combination PDT on tumor growth and regression. These cells were implanted in mammary breast fat pads of nude mice and tumor growth was followed non-invasively by IVIS. The bioluminescent images were captured on: (1) a day after implantation; (2) prior to PDT (pre-PDT); (3) one-week and (iv) two-week post PDT. Growth/regression after PDT was quantitated using BLUs from individual ROI from images captured at different time points during an experiment. Figure 3A shows representative examples of nude mice carrying 4T1 breast tumors imaged just before, one week, and two weeks after PDT, along with vehicle- or CPBN-only treatment groups that did not receive PDT. The bioluminescence signal (pseudo-colored rainbow scale) represents the radiance (photons/s/cm^2^/sr) emitted from 4T1 tumors due to the reaction between luciferin substrate (delivered via i.p. injection) and luciferase enzyme (expressed in the 4T1 cells).

In the vehicle-only group of mice, the luciferase signal increased continually to 4.6-fold by 2 weeks, reflecting unrestricted growth of 4T1 cells [Figure 3A; top left set of images, and Figure 3B]. In a CPBN-treated group of mice that received only CPBN (600 mg/kg/day) once daily for 3 days, the luciferase signal by week 2 was increased by 13.6-fold as compared to the pre-PDT signal [Figure 3A, top right set of images, and Figure 3B]. The ~4-fold induction in growth of 4T1 tumors following CPBN treatment, relative to the vehicle only group, was an unanticipated observation. CPBN, a chemotherapeutic drug approved for the treatment of BCA in humans with anti-proliferative physiological effects, should have reduced the tumor growth, but we note that the incongruent finding might possibly be due to a smaller number of mice (*n* = 4) in this particular treatment group. In the vehicle + PDT group, the rise in bioluminescent signal (2.5 and 9-fold over baseline at weeks 1 and 2, respectively) reflected continued growth and inadequate response of 4T1 tumors to PDT alone [Figure 3A, left bottom set of images, and Figure 3B]. In contrast, the last group of mice that received CPBN + PDT was the only treatment group that showed no tumor growth [Figure 3A, right bottom set of images, and Figure 3B, fourth set of bars]. Thus, the combination of CPBN with PDT showed a much better therapeutic response than PDT alone. Our observation of tumor bioluminescence was only possible up to two weeks post-treatment. At weeks 3 and 4, the signal was often blocked or reduced due to a combination of crust on the dying tumor, increased tumor size, and probably uneven uptake of luciferin due to tumor hypoxia and cell death. We are currently studying the mechanistic details of these effects, with special interest in regulation of apoptotic cell death following CPBN-PDT.

### Distant 4T1 metastases: bioluminescent, histological and immunohistochemical analyses after PDT treatments

**Figure 4.**
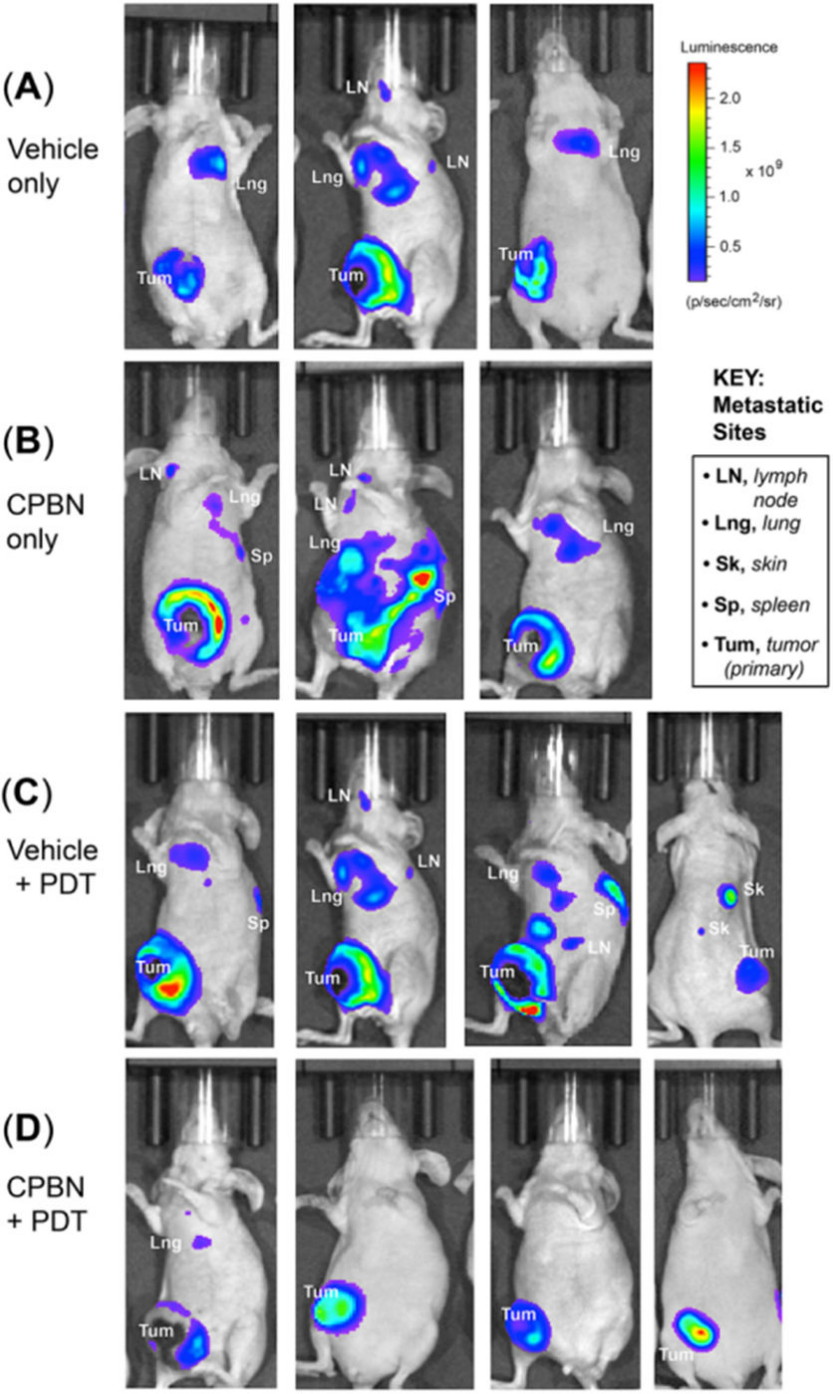
Monitoring of distant metastases of breast tumor cells in nude mice treated with conventional or combination photodynamic therapy (PDT). 4T1 murine breast carcinoma cells were implanted in the breast fat pad of nude mice, and distant metastases were monitored by bioluminescence signals originating from metastatic sites using an in vivo imaging system instrument. Metastatic spread of 4T1 tumor cells (3 weeks post PDT) in mice representing (A) vehicle only; (B) capecitabine (CPBN) only; (C) vehicle + PDT; and (D) CPBN + PDT are shown. Observed organ sites of metastases, including lymph nodes (LN), lung (Lng), spleen (Sp) and skin (Sk) are indicated. Note the significant reduction in the incidence of metastases in CPBN + PDT treated mice

The 4T1 BCA line used in this study has been shown to metastasize widely to distant organs (lymph node, lung, liver, bone, spleen and skin) at later stages of tumor development^[40–42]^. Here we investigated the effect of CPBN-PDT on distant metastases in nude mice, using luciferase-expressing 4T1 cells implanted at a single site on the animal. Metastatic spread to lung, lymph nodes, spleen and skin was observed at three weeks after implantation of the primary tumor site into the ventral breast fat pads [Figure 4]. In addition to the obvious signal at the site of injection, 4T1 cells were also observed to spread in the abdominal cavity and at other distant sites, reflecting likely metastases of tumor cells to lungs, lymph nodes and spleen [Figure 4]. Signals from some specific anatomical locations corresponded to cervical, axillary, sciatic and inguinal lymph node sites. Cutaneous metastases were also seen both visually on the skin (data not shown), and confirmed by IVIS imaging that revealed luminescent signals originating from cutaneous metastatic lesions [Figure 4C, rightmost mouse].

**Figure 5.**
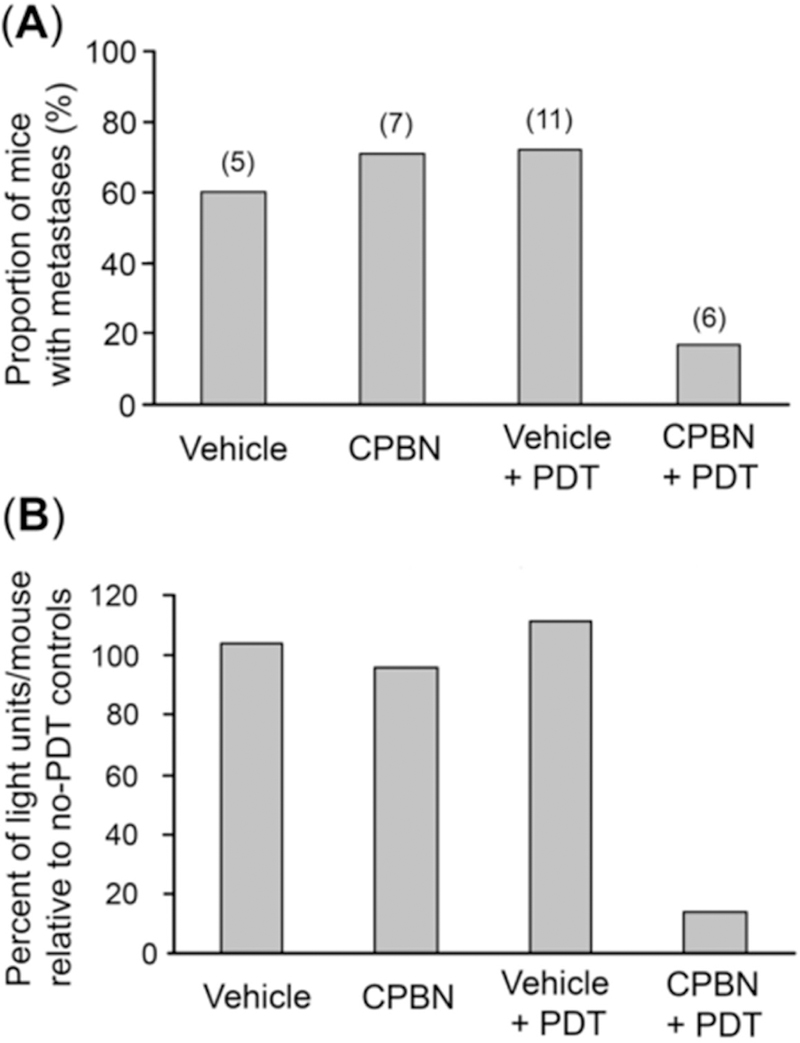
Analyses of incidence of metastases, and total metastatic load, in nude mice with 4T1 breast tumors. Mice from four different treatment groups [vehicle only, capecitabine (CPBN) only, vehicle + photodynamic therapy (PDT) and CPBN + PDT] were quantitatively analyzed as follows. (A) Incidence of metastases by week 3. Note that the first three treatment groups showed 60%−70% of mice with metastases, whereas the CPBN + PDT treatment group showed only 17% of mice with metastases; (B) metastatic tumor load per mouse, calculated by comparing the cumulative bioluminescence units (BLUs) from each mouse with the average cumulative BLUs from no-PDT controls. The metastatic load of 14% in CPBN + PDT group is much lower than the other three groups (96%−111%)

To analyze the effect of combination therapy upon BCA metastases, mice from different treatment groups were examined for the incidence of distant metastases. The mice from three treatment groups, i.e., vehicle, CPBN and vehicle + PDT showed a similar incidence of metastases ranging from 60%−65% [Figures 4A-C and 5A]. By contrast, mice from the CPBN + PDT treatment group showed significantly less metastatic spread [Figure 4D], corresponding to only a 17% incidence of metastases [Figure 5A]. The metastatic tumor load among mice from different treatment groups was compared by analyzing cumulative bioluminescence originating from different ROIs on each mouse. Mice from vehicle-only, CPBN-only, PDT-only treatment groups, showed a similar metastatic load [Figure 5B]. However, the CPBN + PDT treatment group showed a significantly reduced metastatic load of only 14% [Figure 5B].

**Figure 6.**
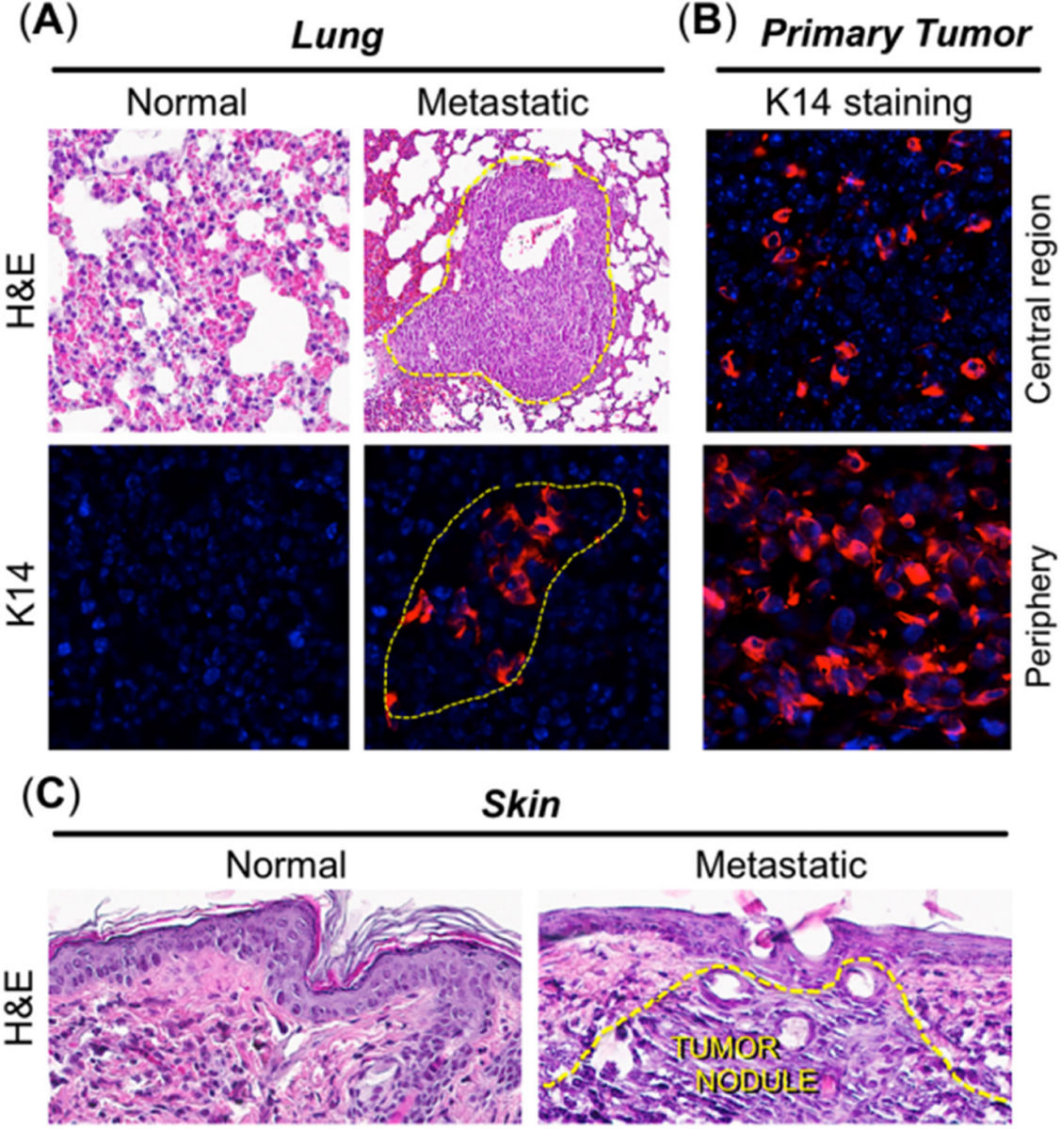
Histological and immunohistochemical analyses of distant metastases in the 4T1 breast tumor model. Tissue biopsies from organs positive for bioluminescence, along with primary tumors, were harvested three weeks after photodynamic therapy. By histological analyses, metastases were detectable in lungs and skin only. (A) Metastatic nodules were visible in lungs via hematoxylin and eosin (H&E) staining, and by immunostaining with antisera to keratin 14 (K14; red color), as delineated by dotted yellow lines; (B) positive K14 staining in primary breast tumor, to confirm that lung tumor nests originated from the primary breast tumor. K14 staining was weaker in the tumor center (top panel) than in the tumor periphery (bottom panel), possibly due to hypoxia and necrosis in the center *vs.* proliferation at the tumor periphery; (C) a cutaneous metastasis, detected visually and by *in vivo* imaging system, revealed obvious infiltrating tumor cells when stained with H&E (right panel, dotted yellow lines)

To further confirm that the bioluminescence observed in Figure 4 did indeed represent tumor metastases, various organs were necropsied and stained with hematoxylin and eosin. Our histological and immunohistochemical analyses were only able to reveal 4T1 metastases in the lungs and skin, possibly due to the difference in sensitivity of detection between IVIS and hematoxylin and eosin methods. IVIS is an *in vivo* optical imaging system capable of detecting signal from as fewer as 3 cells implanted in a mouse^[43]^, as compared to a few hundred cells required to form a tumor nest that can be detected histologically by hematoxylin and eosin. Figure 6A clearly shows the presence of metastatic tumor nests (marked with dotted lines) in the lung of a mouse that was positive by IVIS imaging. Keratin 14 (K14, a marker for epithelial breast tumors at ectopic sites; Figure 6A), was expressed only within tumor nests in the lungs of tumor-bearing mice and not in the lungs from control mice [Figure 6A; bottom]. Comparing this to the primary tumor (which also stained strongly for K14; Figure 6B), the pattern of K14 expression was interesting, in that the most intense expression was observed in the tumor periphery, with far fewer positive cells in the central/stromal (hypoxic) tumor regions (Figure 6B; top *vs.* bottom), possibly due to hypoxia and necrosis in the center and the proliferative nature of the tumor periphery, respectively. This observation further supports the assertion that the primary breast tumor was the origin of the K14-positive, metastatic tumor nests in the lungs. Histology of cutaneous metastatic lesions also clearly confirmed the presence of metastatic carcinoma in skin (Figure 6C; righthand image marked with dotted lines). Further exploration of how CPBN-PDT exerts its inhibitory effect on 4T1 metastatic spread is underway.

## DISCUSSION

In this study we have shown, in a murine model of BCA, that pretreatment with CPBN prior to ALA-mediated PDT causes selective enhancement of PpIX levels within 4T1 tumors, and improves the treatment outcome of PDT by enhancing tumor cell death. We also showed, using histological and immunohistochemical analyses, that the CPBN-PDT combination enhances the differentiation state of 4T1 carcinoma cells within the tumors. Finally, using *in vivo* bioluminescent monitoring, we showed that the combination approach appears more effective than ALA-PDT alone, retarding the growth of the primary tumor, and also reducing metastatic spread.

The observed beneficial effect of neoadjuvant CPBN on the therapeutic outcome of PDT may involve several underlying mechanisms that we previously identified in studies with other differentiation-promoting drugs such as methotrexate, vitamin D and 5-FU^[9]^. The first apparent mechanism, an inhibition of tumor cell proliferation [Figure 2B and F], is an anticipated effect of 5-FU since the latter inhibits TS and induces growth arrest by mis-incorporation of FU into DNA^[38]^. The second mechanism, enhanced differentiation of tumor cells, is a less understood effect whose basis is unclear since the induction of growth arrest is often associated with induction of differentiation. The third mechanism, enhancement of PpIX accumulation, is actually now fairly well understood from our previous work^[24]^. PpIX is produced by enzymatic conversion of ALA through the heme biosynthetic pathway. 5-FU, the final product of CPBN metabolism, has been shown to exert its effects on PpIX levels in murine and human SCC tumors by upregulating the levels of two rate-limiting enzymes of the heme pathway, coproporphyrinogen oxidase (upregulation) and ferrochelatase (down regulation), in a way that favors accumulation of higher PpIX levels inside the tumor cells’ mitochondria^[24]^. The ultimate consequence of elevated PpIX levels following CPBN pretreatment, is a significant increase in tumor cell death after PDT. This is due not only to higher PpIX concentrations within individual cells, but also to a more homogeneous distribution of high PpIX throughout the tumor [Figure 1C]. Following PDT, the loss of tumor cells through apoptosis was demonstrated via TUNEL and hematoxylin and eosin staining; thus, apoptosis clearly contributes to better outcomes such as retardation of tumor growth in the CPBN-PDT treatment group. Our data regarding inhibitory effects of the CPBN-PDT regimen upon metastases are particularly exciting. Although our sample sizes were relatively small and the data should be considered somewhat preliminary, the CPBN-PDT combination appears to be more effective at preventing metastatic spread than either CPBN or PDT alone [Figures 4 and 5].

The clinical implication of this study is the possibility that a new combination approach might bring PDT closer to the forefront of options available for management of BCA. Mainline treatment options available for BCA today include RT, surgery, chemotherapy, biological therapy (targeted agents), and hormonal therapy. As approaches to BCA disease management have evolved, the focus has shifted toward breast preservation, placing less emphasis on surgical and ablative approaches. In this setting, PDT, represents a promising treatment and management strategy for the following reasons: (1) Unlike RT, PDT does not cause fibrosis and scarring; (2) PDT can be repeated many times since it targets mitochondria rather than DNA, and thereby poses minimal risk for inducing mutations and secondary tumors; (3) PDT has already been successfully tried as a means to control recurrent BCA, especially for recurrences on the chest wall (which are extremely resistant to chemotherapy for unknown reasons). Thus, a recent clinical trial from China used ALA-mediated PDT in combination with RT for BCA metastases of the chest wall, and showed that the alternating use of PDT and RT improved the complete response rate, and reduced the time to clearance, when compared to RT alone^[19]^. Increased use of PDT as a tissue-sparing adjunct for RT, would reduce the amount of radiation-induced fibrosis and vascular damage (scarring, ulceration, dermatitis) suffered by BCA patients, thereby representing a meaningful advance in clinical oncology; (4) it might be possible to boost the efficacy of PDT, by using neoadjuvantal CPBN, to the extent that CPBN-PDT becomes useful as a monotherapy. At the very least, CPBN-enhanced PDT would be more effective as an RT-sparing approach when used together with RT; (5) future clinical translation of a CPBN-PDT approach is likely to be relatively easy, since CPBN (trade name Xeloda^®^) is already a well-established, FDA-approved agent for the treatment of metastatic BCA.

In conclusion, based on our previous work showing that a combination of 5-FU given prior to PDT, leads to enhanced ALA-mediated PpIX levels and greater tumor cell death following PDT in several different epithelial cancers, we hypothesized here that CPBN might be a useful alternative to 5-FU as a neoadjuvant with PDT for BCA. Because 5-FU might be quite toxic at the high systemic levels required to modulate PpIX levels in BCA tumor, we reasoned that CPBN, a precursor to 5-FU, might serve as a safer yet equally effective alternative. In this study, we compared the responses of 4T1 tumors when treated with either conventional ALA-PDT, or with ALA-PDT preceded by oral CPBN. Our results revealed the following major findings: (1) CPBN-enhanced PDT can be used to significantly increase the accumulation of PpIX in murine breast tumors, and therefore represents a non-toxic alternative to 5-FU; (2) the pretreatment of 4T1 tumors with CPBN causes increased differentiation and decreased proliferation; (3) CPBN-enhanced PDT improves photodynamic killing of the breast tumor cells; (4) a combination regimen using CPBN and PDT significantly reduces distant metastases in the 4T1 tumor model. In summary, this treatment combination has the potential to be an effective therapy for BCA metastases, and potentially for other types of cancer. A more extensive version of this preclinical study would be immensely exciting and provide an additional rationale for designing a clinical trial based upon this concept.
